# The Plasma Membrane Potential and the Organization of the Actin Cytoskeleton of Epithelial Cells

**DOI:** 10.1155/2012/121424

**Published:** 2012-01-23

**Authors:** Silvia Chifflet, Julio A. Hernández

**Affiliations:** ^1^Departamento de Bioquímica, Facultad de Medicina, Universidad de la República, Gral. Flores 2125, 11800 Montevideo, Uruguay; ^2^Sección Biofísica, Facultad de Ciencias, Universidad de la República, Iguá 4225 esq. Mataojo, 11400 Montevideo, Uruguay

## Abstract

The establishment and maintenance of the polarized epithelial phenotype require a characteristic organization of the cytoskeletal components. There are many cellular effectors involved in the regulation of the cytoskeleton of epithelial cells. Recently, modifications in the plasma membrane potential (PMP) have been suggested to participate in the modulation of the cytoskeletal organization of epithelia. Here, we review evidence showing that changes in the PMP of diverse epithelial cells promote characteristic modifications in the cytoskeletal organization, with a focus on the actin cytoskeleton. The molecular paths mediating these effects may include voltage-sensitive integral membrane proteins and/or peripheral proteins sensitive to surface potentials. The voltage dependence of the cytoskeletal organization seems to have implications in several physiological processes, including epithelial wound healing and apoptosis.

## 1. Introduction

The transport of water and solutes across epithelial layers represents a major achievement of biological evolution and constitutes the basis for the existence of higher organisms [1]. To accomplish their transport properties, the epithelial cells acquire characteristic structural and functional features. An epithelial layer must constitute a well-defined macroscopic permeability barrier, which results in the selective transport of solutes and water across the overall tissue. For this, transport epithelia must develop a complex set of cell junctions and a polarized distribution of membrane molecules, which localize at distinct apical and basolateral domains of the plasma membrane [2–5].

The establishment and maintenance of the polarized epithelial phenotype require a characteristic organization of the cytoskeletal components. There are many cellular effectors involved in the regulation of the cytoskeleton of epithelial cells [6–11]. Recently, modifications in the plasma membrane potential (PMP) have been shown to participate in the modulation of the cytoskeletal organization. The purpose of this paper is to review evidence relating PMP modifications to changes in the cytoskeletal organization of epithelia, with an emphasis on the actin cytoskeleton, and discuss possible molecular paths mediating these effects. Prior to this, we briefly review the basic characteristics of the cytoskeleton and the generation of plasma membrane potentials in epithelial cells.

## 2. General Morphological Aspects and Organization of the Cytoskeleton of Epithelial Cells

**Figure 1:**
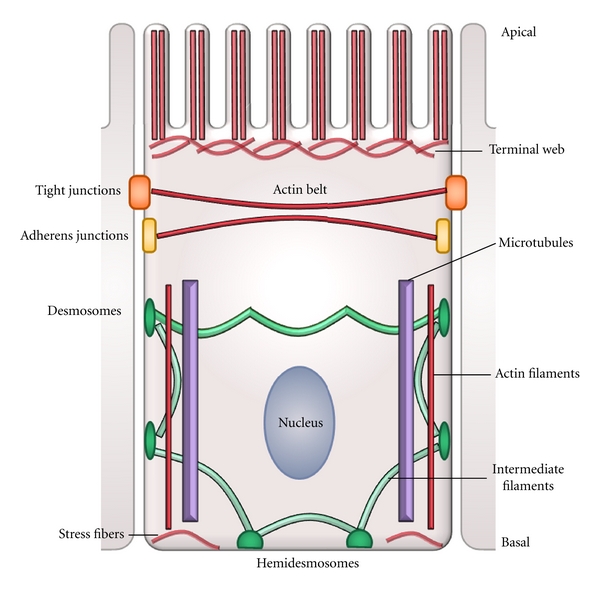
Schematic representation of the cytoskeletal organization of a transporting epithelial cell. The scheme shows the cell-cell and cell-substrate junctions connecting the actin, tubulin, and intermediate filament cytoskeletons. See text for details.

**Figure 2:**
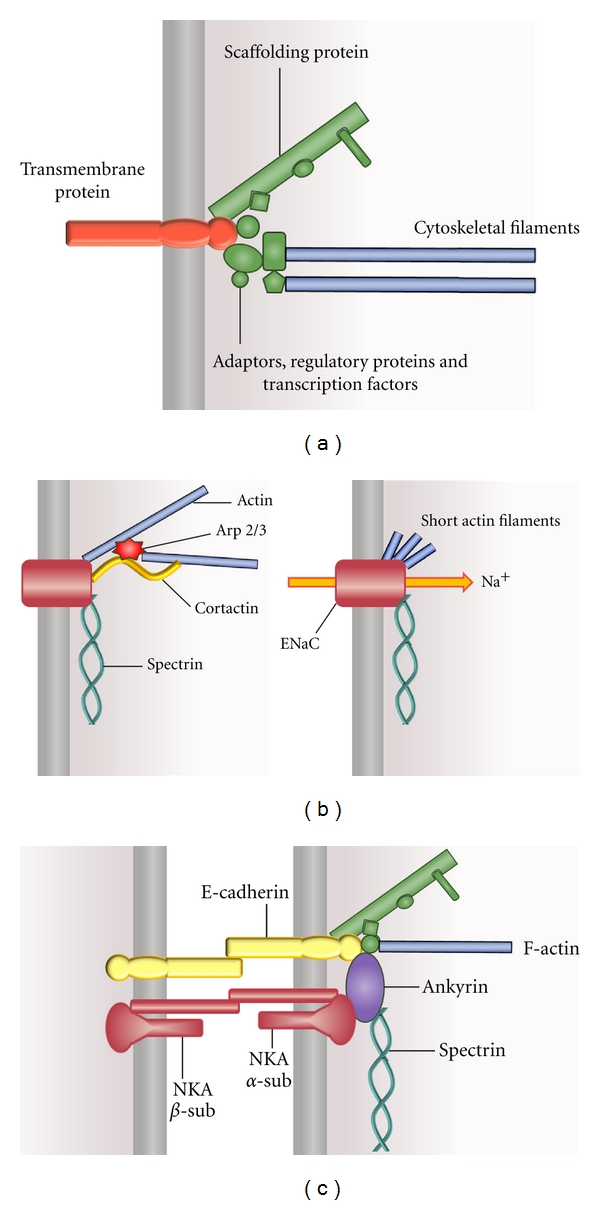
(a) General schematic representation of an anchoring junction. The scheme depicts the basic organization of the proteins comprising an anchoring junction. The three organizational levels (see text) are represented by different colors; modified from Matter and Balda [34]. (b) Scheme representing some of the interactions between ENaC and the cytoskeleton. The channel is kept in its membrane location via the spectrin-based cytoskeleton. In the nonstimulated state, ENaC is bound to F-actin directly and/or by cortactin-Arp2/3 interaction (left panel). Diverse stimuli promote formation of short actin filaments that activate ENaC by direct binding to the channel (right panel). See main text for references. (c) Scheme representing some of the interactions between the Na^+^-K^+^ ATPase (NKA) and the cytoskeleton; modified from Bennett and Healy [18].

Mature epithelia are characterized by two fundamental morphological and functional features: a tight cellular packing, supported by the existence of strong adhesive forces between neighboring cells and a polarized cellular phenotype [5]. These two properties are interdependent, since the establishment of intercellular junctions represents the main positional cue triggering the development of cell polarization. All the anchoring junctions, either between cells or between cells and substrate, are associated to cytoskeletal components that are crucial for the junction stability. The characteristic organization of the cytoskeleton of epithelial cells greatly depends upon these interactions with the cell junctions, as depicted in Figure 1. In particular, the actin cytoskeleton associates with diverse cellular structures in mature epithelial cells (Figure 1). In these cells, the most conspicuous actin structure is the circumferential actin belt, a bundle of actomyosin fibers located immediately beneath and associated with the tight and adherens junctions. Here, the microfilaments and the cell junctions interact via a complex set of multifunctional proteins (Figure 2(a)). Actin filaments can also be found along the lateral membrane [12–14], where it codistributes with myosin I [15–17]. Short actin filaments are part of the spectrin-based membrane skeleton network. Besides its classical role in membrane domain organization [18], this network is involved in the biogenesis of the lateral membrane of epithelial cells and in the maintenance of their columnar shape [19, 20]. Stress fibers at the basal domain are also actomyosin structures associated to focal contacts and other cell-substrate junctions [21]. At the apical domain, parallel bundles of crosslinked actin filaments, extending from the terminal web, constitute the core of the microvilli.

Polarized epithelial cells exhibit a characteristic pattern of microtubule organization (Figure 1) [6, 22, 23]. Unlike fibroblastic-like cells, where centrosomes are responsible for the nucleation and organization of microtubules, in epithelial cells, most microtubules are acentrosomal. They also present distinctive properties in their behavior, stability, dynamics and regulation [24–27]. Typically, in epithelia microtubules organize in long apicobasal-oriented parallel fibers that span the whole length of the cell, with their minus end pointing towards the apical surface and in disordered short filament networks underneath the apical and basal membranes [28–31]. This particular arrangement of microtubules is crucial for the targeted traffic of vesicles that sustains the existence of distinctive apical and basolateral domains of epithelial cells [23, 32]. Figure 1 also schematically shows that the other major component of the cytoskeleton, the intermediate filaments, traverses epithelial cells connecting desmosomes and hemidesmosomes [33].

Intercellular anchoring junctions are key structures for epithelial organization and function. Among these, the tight junctions (zonula occludens) selectively seal the intercellular space at the apical side and prevent the exchange of membrane proteins and lipids between the apical and basolateral domains. Underneath the tight junctions, the adherens junctions (zonula adherens) and desmosomes are the main responsible for the mechanical strength of the cell-cell contacts. A schematic description of the structure of anchoring junctions is depicted in Figure 2(a). As emphasized by Matter and Balda [34] for the case of tight junctions, three levels of organization can be recognized. This conception can be generalized to all the anchoring junctions. The first level corresponds to the integral membrane proteins, the second to a set of proteins mediating, among others, the interaction between the cytoskeleton and the adhesion proteins, and the third to the cytoskeletal proteins. The second level is made up of a complex array of soluble proteins, with many of which being capable to transit between other cellular compartments and their juxtamembrane location at the corresponding cell junction [33]. This complex includes transcription factors, regulatory proteins, and scaffolding proteins and is a critical site for the transduction of diverse types of signals [35]. Among these, the acquisition of the epithelial phenotype is triggered by the recruiting of proteins at the second and third junction levels, initially induced by the establishment of cell-cell and cell-substrate contacts [36]. The composition and interactions of the proteins of the second level are highly complex and still remain matters of active investigation. The general organizational pattern shown in Figure 2(a) for the anchoring junctions can also be extended to include the linking between other types of integral membrane proteins, such as ion channels and pumps and the cytoskeleton (see below).

Adherens junctions (AJs) have a central role in the establishment and maintenance of the epithelial phenotype. In this respect, classical cadherins have been recognized to be involved in the establishment of early intercellular contacts and in the organization of microfilaments and microtubules [8, 11, 36]. The association between AJ and these cytoskeletal components is interdependent, since the actin and tubulin cytoskeletons in turn contribute to AJ formation, stability and strength [37–43]. Thus, when actin cytoskeleton is disrupted, AJ formation is impaired [38, 39, 44]. Moreover, actin cytoskeletal reorganization, as induced for instance by extracellular calcium deprivation, may determine AJ and tight junction disruption with the consequent loss of the epithelial integrity [45, 46]. Likewise, cell-cell contacts stabilize the plus and minusends of microtubules at the adherens junctions, while the blocking of their dynamic turnover provokes brakeage of cell-cell contacts [26, 43, 47]. 

 Even in the quiescent state, the cytoskeleton of epithelial cells is highly dynamical, undergoing constant assembly and disassembly of its structural units. For this reason, it is subject to complex mechanisms of regulation, many of which involve components of cell-cell junctions and other membrane proteins. Since it is not the purpose of this work to make a thorough revision of this rather involved issue, the reader is referred to several specialized reviews [11, 36, 48, 49]. In Section 5 we will only mention some regulatory pathways that are possible candidates to mediate the responses of the cytoskeleton to modifications in the plasma membrane potential.

## 3. Interactions between Ionic Transport Systems of the Plasma Membrane and the Cytoskeleton

Besides its associations with the cell junctions, the cytoskeleton also interacts, directly or indirectly, with diverse ion transport systems of the plasma membrane [50–53]. Similarly to the case of the cell junctions, many of the associations of the cell membrane channels and transporters with the cytoskeleton are interdependent (for detailed reviews, see [50, 52]). In this way, several transport systems are anchoring sites for the cortical cytoskeleton [54–62]. Conversely, the binding to the cytoskeleton modulates diverse ion transport activities [56, 61, 63–68]. This interrelation between the cytoskeleton and ionic transport systems plays a relevant role in the physiological properties of transport epithelia [69, 70]. For example, in the renal medulary thick ascending limb the Na^+^/H^+^ exchanger regulates bicarbonate absorption by controlling the organization of the actin cytoskeleton [71]. In human syncitiotrophoblast, gelsolin (an actin regulatory protein) stimulates nonselective cation channels of the TRP family in the presence of calcium [72]. In HEK 293 cells, calcium-activated chloride channels require cytoskeletal interactions to achieve full activation [73]. A most noteworthy example of the modulation of ionic transport by the cytoskeleton is provided by volume-sensitive ion transport systems, that modify transport rates in response to changes in cytoskeletal tension [74–76]. In several epithelial cells, where water and salt transcellular transport determine modifications in the cellular volume, stretch-activated potassium and chloride channels participate in the regulation of salt transport [77–79]. Among other examples of the regulation of ionic conductances by the cytoskeleton, in mammary adenocarcinoma cells a well-organized actin network is necessary for the proper activation of CFTR by cAMP [80].

The interrelationship between ion transport systems and the cytoskeleton is also crucial in cell adhesion and migration, processes requiring significant cytoskeletal remodeling and modifications of ion transport. In this respect, it has been shown that inhibition of the CFTR-dependent conductance impairs lamellipodia formation in bronchial epithelial cells [81]. In T-cells, Kv1.3 channels participate in promoting adhesion by establishing complexes with *β*1 integrin [82]. In neuroblastoma cell lines, TRPM7 channels affect cell adhesion by direct interaction with the actomyosin cytoskeleton [83]. TRPV4 has also been reported to form complexes with the actin cytoskeleton and regulatory kinases, involved in lamellipodial formation [61]. Focal adhesion kinase, an enzyme involved in integrin-mediated focal adhesion, is activated by forming complexes with Kv1.2 channels both in epithelial and nonepithelial cells [84]. The inhibition of either the Na^+^/H^+^ exchanger transport activity or its actin cytoskeletal anchoring significantly decreases migration of PS-120 fibroblasts [66]. Also, ENaC has been reported to participate in the processes of cell migration and wound healing in epithelia [85, 86] and other tissues [87]. The molecular nature of this role may imply direct interactions between this channel and cytoskeletal components. In this respect, it has been shown that the alpha subunit of ENaC associates with spectrin [54, 88] and short actin filaments [89]. In addition, there is evidence that the direct binding between ENaC and these filaments modifies the channel conductance [90–92]. More recently, Ilatovskaya et al. [68] have shown that, in renal epithelial cells, the actin cytoskeleton regulates ENaC activity via a cortactin-Arp2/3 complex. As an example of the structural relationships between an ion channel and the cytoskeleton, Figure 2(b) resumes diverse evidence in a schematic diagram of the interactions between ENaC and cytoskeletal components during nonactivated and activated conditions [50, 54, 68, 88]. To be noted, the mechanism through which the cytoskeleton promotes modifications in ionic conductance may depend on the particular transport system. For the case of ENaC, Figure 2(b) shows that the channel activation requires direct binding to short actin filaments, produced by direct PKA phosphorylation of actin or via an actin regulatory protein [50]. As another example, in vascular endothelia 4.1 proteins have been proposed to be necessary for the stable expression of TRPC4 in the plasma membrane [93].

The sodium pump has also classically been recognized to establish interactions with the cortical cytoskeleton [94]. More recently, it was shown that this enzyme participates in tight junction assembly in MDCK cells via activation of RhoA and stress fiber formation [95–97]. In caveolar structures the sodium pump is the core of a multiprotein complex, the sodium pump signalosome, that contains several proteins involved in cytoskeletal regulation [98]. Moreover, in vitro experiments reinforce the existence of interrelations between the Na^+^-K^+^-ATPase and actin [64]. The molecular nature of the interactions between the sodium pump and the cytoskeletal components, and their physiological relevance, are beginning to be known in more detail. In this respect, the critical role of the interaction of ankyrin-G and the spectrin-based actin cytoskeleton in the membrane retention of the sodium pump has been well established [19, 99]. The binding of ankyrin with the *α*_1_-subunit of the Na^+^-K^+^ ATPase has been shown to be crucial not only for the membrane anchoring of the enzyme, but also for its trafficking in the polarized cell [100]. Based upon the available evidence, the scheme of Figure 2(c) summarizes some of the recognized interactions between the sodium pump and the cytoskeleton in vertebrate cells. In *D. melanogaster*, Dubreuil and coworkers demonstrated the existence of ankyrin-independent interactions between the sodium pump and spectrin [101, 102].

## 4. The Plasma Membrane Potential of Epithelial Cells

**Figure 3:**
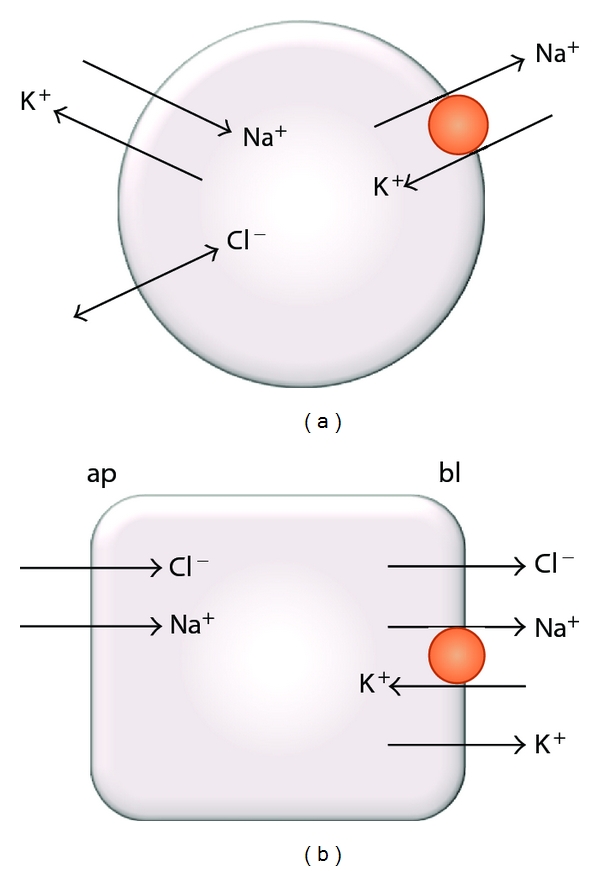
Schemes showing some ionic transport systems of the plasma membrane. (a) In symmetric, nonpolarized cells the sodium pump maintains the electrochemical gradients of sodium and potassium across the plasma membrane. The sodium and potassium channels underlie the generation of a diffusion potential across the plasma membrane. Chloride is usually maintained at activities close to equilibrium. (b) In polarized epithelial cells the asymmetric distribution of, mainly, sodium channels and the sodium pump into distinctive apical and basolateral membranes may determine a net transcellular transport of sodium chloride. (ap: apical; bl: basolateral; the orange circles represent the sodium pump, and the other arrows correspond to ionic channels).

The classical dogma of the ionic transport properties of epithelia was founded by Ussing and coworkers in the 1950s and has ever since become the basic paradigm of epithelial transport [103–105]. According to this model, in its essential terms, the polarized distribution of the sodium pump and sodium channels is the basic process that transforms a homogeneous cell into an epithelial cell, capable of performing net transepithelial transport of salt between two separated compartments (Figure 3). Although the general scheme of Figure 3(b) may apply to different epithelial cell types, it is particularly characteristic of tight epithelia [106]. In this respect, it was recognized that epithelia can be divided into two categories, tight and leaky, according to the electrical resistance of the tight junctions [107]. In addition to this difference, the two types are distinguished by other transport properties. Thus, besides the low electrical resistance, leaky epithelia also exhibit a high water permeability and establish low-transepithelial-potential differences (see below), while the reverse is true for tight epithelia [108].

In general, since the apical and basolateral domains of epithelial cells have different compositions of ionic transport systems, the mechanisms of generation of the membrane potential differ between them. For the case shown in Figure 3(b), the PMP across the basolateral membrane is approximately given by a diffusion potential dominated by potassium. Among other differential contributions, the sodium pump may be responsible for the generation of the basolateral PMP in an electrogenic fashion, particularly if potassium permeability is low [109]. The composition of the apical membranes greatly varies among the different epithelial cell types. Therefore, the generation of the apical PMP depends on the specific epithelia considered. For example, in intestinal epithelial cells the sodium-glucose electrogenic cotransport may be the major contributor to the apical PMP [110]. The selective modifications in the ionic conductances at the apical or basolateral domains may play physiological roles in diverse epithelia. Thus, in the acinar cells of parotid salivary glands, modifications in the potassium conductance of the apical membrane produce changes in the fluid flow via changes in the apical PMP [111–113]. In pancreatic acinar ducts, modifications in the apical depolarization mediated by the sodium-glucose cotransporter modulate the amount of basolateral uptake of chloride and bicarbonate [114]. Another example is given by the basolateral membranes of tracheal and intestinal cells, where potassium channels containing the *KCNE* family of *β*-subunits control the chloride flux via modifications in the basolateral PMP [115].

**Figure 4:**
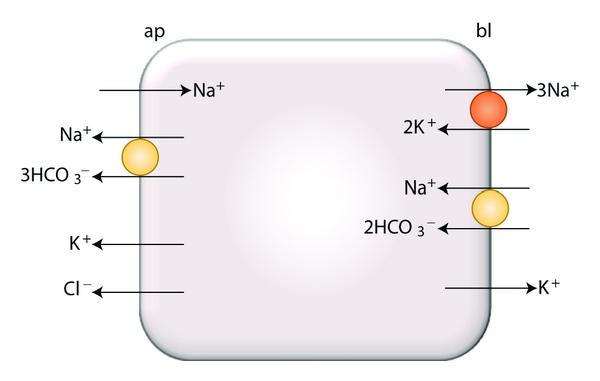
Scheme of a corneal endothelial cell showing electrogenic transport systems of the apical (ap) and basolateral (bl) membranes. Orange circle and arrows as in Figure 3, pale orange circles represent sodium-bicarbonate cotransporters; modified from Montalbetti and Fischbarg [117].

In epithelial cells, the differential generation of the PMP at the two membrane domains determines the existence of a net transepithelial potential difference (TPD). As mentioned above, tight epithelia are capable to maintain large TPDs as a consequence, among other factors, of the existence of a large electrical resistance of the paracellular pathway [108]. On the contrary, the TPDs across leaky epithelia are generally small. As an example, Figure 4 shows a scheme of some of the ionic transport systems of corneal endothelium, a typical leaky epithelium that pumps salt and water from the corneal stroma to the aqueous compartment of the eye [116, 117]. As can be seen, the presence at the two domains of sodium-bicarbonate cotransporters with different stoichiometric ratios is the cause for a small TPD (i.e., less than 1 mV). Among other possible roles, as in paracellular ion movement, this TPD may have a relevant function in the mechanism of solute-solvent coupling across corneal endothelium [118].

Epithelial cells in culture may acquire some structural and physiological characteristics of the in situ epithelial cells, including the generation of a TPD [119–121]. For the case of small TPDs, as is the case of corneal endothelium [116], the employment of voltage-sensitive fluorescent probes provides with global PMP values which are approximately equal to the apical and basolateral PMPs of these cells in culture [117, 122] but does not permit to distinguish between them. Electrophysiological procedures are required to determine the PMP at each one of the plasma membrane domains, both under in situ or culture conditions.

## 5. Modulation of the Epithelial Actin Cytoskeleton by Modifications in the Plasma Membrane Potential

Several authors have suggested that the plasma membrane potential of nonexcitable cells could play a role in diverse cellular processes [123–125]. In MDCK cells, Vaaraniemi et al. [126] found that activation of protein kinase C determined PMP depolarization and reorganization of the spectrin-based and actin cytoskeletons. Consistently with these findings, we showed that the nonspecific modifications of the PMP (i.e., depolarization or hyperpolarization) promote changes in the organization of the actin and tubulin cytoskeletons in bovine corneal endothelial (BCE) cells in culture [127, 128]. In particular, the changes observed for the actin cytoskeleton consisted, for the PMP depolarization, in a gradual loss of the peripheral ring, an increase in F-actin throughout the cytoplasm, appearance of intercellular gaps and, for sufficiently prolonged treatments, eventual cell detachment [127]. Conversely, it was noteworthy to confirm that PMP hyperpolarization determined the opposite response, that is, an increase in the compactness of actin at the peripheral ring and an augmented resistance of intercellular adhesion to diverse destabilizing procedures [128]. These cytoskeletal responses to PMP modifications were characteristic of some cultured epithelia in confluence displaying a typical epithelial phenotype exhibiting, among other characteristics, a well-defined circumferential actin ring, whereas nonconfluent or undifferentiated epithelial cell lines did not manifest a recognizable response [128, 129]. Effects of PMP depolarization on microfilaments were also observed in kidney tubular cells, where the authors demonstrated that Rho activation and the consequent increase in phosphorylation of the light myosin chain are involved [130, 131]. The role of the PMP on the cytoskeletal organization was also supported by the finding that, in vascular endothelia, depolarization decreases cell stiffness by affecting the cortical actin cytoskeleton [132, 133].

The activities of diverse signaling intermediates are sensible to modifications of the PMP. Thus, in excitable cells, regulators of G protein signaling (RGS), Rho proteins and PKA are activated by the calcium increase provoked by PMP depolarization [134–140]. In renal epithelia, however, the activation of Rho determined by depolarization is not mediated by cytosolic calcium increase [130, 141]. Among the integral membrane proteins that could mediate cytoskeletal responses to diverse effectors, the phosphatidylinositol phosphatase Ci-VSP [142] and its homologs [143, 144] contain a voltage sensor in the transmembrane domain [145] and produce PIP2, a well-known regulator of the actin cytoskeleton [146, 147]. Interestingly, this enzyme, present in epithelial and nonepithelial cells [143, 144], is activated by PMP depolarization [142]. More recently, in *Xenopus* oocytes Zhang et al. [148] described an alternative voltage-sensitive mechanism to increase PIP2 level in response to PMP depolarization, via activation of a PI4 kinase. The G protein-coupled receptors (GPCR) constitute another family of membrane proteins shown to initiate signaling paths leading to actin remodeling [149, 150]. These receptors, activated by a variety of extracellular effectors, are directly regulated by the PMP [151–156].

At this point, it should be reminded that the “plasma membrane potential,” as determined from typical electrophysiological procedures, refers to net electrical potential differences between the intra- and extracellular bulk compartments. This difference comprises a series of intermediate electrical potential changes that include surface potentials at membrane proximities and the transmembrane potential [123, 157, 158]. Changes in the surface potentials could affect peripheral proteins, many of them involved in cytoskeletal regulation [123, 158]. For example, diverse putative peripheral proteins can bind to the inner surface of the plasma membrane by electrostatic interactions and modify their degree of attachment in response to modifications in the surface potential, such as MARCKS [159], PTEN [160], K-Ras [161], c-Src: [162], Rac1 [163], and ERM proteins [164]. For the particular case of K-Ras it has been shown that, apart from the inner surface potential, the transmembrane potential also affects its binding to the plasma membrane [165].

The effects of the PMP on the cytoskeletal organization could also be mediated by membrane ionic transport systems directly or indirectly connected to cytoskeletal elements, such as the ones mentioned in Section 3. In principle, there are two main mechanisms through which the PMP could affect the interrelationship between ion transport systems and the cytoskeleton: (a) by modifying ionic currents and thus the ionic environment near the cytoskeletal binding regions, and (b) by determining electroconformational modifications of the ion transporting proteins that can be propagated to the cytoskeletal components, as could be the case for other nontransporting integral membrane proteins. As an example of this latter possibility, several ion channels have been proposed to transmit signals via conformational coupling with integrins, irrespectively of changes in the ionic fluxes [166]. Whatever the mechanism, it must be noted that the particular ion transport system that would mediate the cytoskeletal response to a certain PMP modification (i.e., a hyperpolarization or a depolarization) may depend on the specific procedure employed for the modification. For instance, in a certain cell type PMP hyperpolarization may be achieved by increasing the potassium conductivity, determining augmented potassium efflux, or by increasing the chloride conductivity, producing increased chloride influx. Correspondingly, the intermediate path and specific organizational response of the cytoskeleton to the particular PMP change provoked may also depend on the specific procedure and ionic path (cf. Section 3).

Another possible mechanism mediating the effects of PMP modifications on cytoskeletal rearrangements could be the direct conduction of electrical signals generated at the plasma membrane by the cytoskeletal components themselves. In support of this idea is the finding that actin filaments can propagate electrical signals per se [167]. 

## 6. Possible Physiological, Pathological, and Medical Implications of a Regulation of the Actin Cytoskeleton by the Membrane Potential

In principle, it could be expected that the transition of an epithelial cell from a quiescent to a specific secretion or absorption state occurs with characteristic modifications both in cytoskeletal organization and ionic conductances. As suggested from the evidence reviewed in this work, these concurrent modifications could be mediated by the complex regulatory framework provided by the interactions between cytoskeletal components and diverse membrane transport systems. From the results reviewed in the previous section the modifications in the plasma membrane potential could participate in the regulation of the organization of the cytoskeleton of epithelial cells, possibly via effects mediated by ionic transport systems. The examples shown in Section 3 support this notion by describing diverse examples of epithelial cells where ionic transport is associated to cytoskeletal modifications.

As a further physiological counterpart to the results commented in Section 5, we put into evidence that PMP depolarization occurs during wound healing in bovine corneal endothelial cells, as a consequence of the increased expression of the epithelial sodium channel (ENaC), and that it may have a role in the healing process [85]. To be noted, the border cells actively participating in the healing response of epithelia experience characteristic reorganizations of the actin cytoskeleton, which are blocked by ENaC inhibition (ibid). ENaC-dependent PMP depolarization was also observed in the course of wound healing by others in a cell line of human trophoblast [86] and by us in other epithelia in culture (unpublished results). Interestingly, in healing corneal endothelium and epithelium Watsky [168] described a late hyperpolarizing potassium current that, in view of the results described above, could have the role of restituting the membrane potential to its basal value. A role for ENaC in the processes of wound healing was also proposed by Grifoni et al. [87] for smooth vascular muscle cells.

Actin has been found to participate in the development of the apoptotic response [169]. Thus, interference with actin dynamics by inhibition of its depolymerization enhances apoptotic activity in HL-60 cells [170, 171]. However, in T-cells disruption of the actin cytoskeleton promotes caspase-3-mediated apoptosis [172]. A concurrent finding of interest within the conceptual framework of this paper is that cells undergo PMP depolarization in the course of apoptosis, a fact that has been speculated to play a role in the cytoskeletal reorganization that takes place during this process [173]. 

 The finding that hyperpolarization of the PMP determines actin compaction along the adherens junctions and increases junction stability [128] may have application in the design of therapeutic strategies. In this respect, the loss of epithelial intercellular adhesions is at the basis of diverse pathologies [174, 175]. Some of these represent major medical challenges, such as cancer progression [176–179] ischemic injuries [180] and bowel inflammatory diseases [181].

## 7. Concluding Remarks

The modifications in the plasma membrane potential have mostly been classically associated with the physiology of excitable tissues. In both excitable and nonexcitable cells, the PMP is an energetic component of the electrochemical gradients responsible of membrane ionic transport. The findings reviewed in this work contribute to the concept that the PMP may also participate in other cellular processes, including the establishment and maintenance of the morphological and functional features of epithelial cells. In particular, we have emphasized here the possible role of the PMP in the regulation of the actin cytoskeleton. Although some knowledge about signaling pathways involved in the transduction of electrical signals at the plasma membrane to mechanical modifications of the cytoskeleton has been unraveled, the involvement of the cytoskeleton in many relevant physiological cellular phenomena permits to anticipate great progress in this respect in the near future.
